# A study of the effects of early diagenesis on the geotechnical properties of carbonate sediments (North West Shelf, Australia)

**DOI:** 10.1038/s41598-024-67207-2

**Published:** 2024-07-20

**Authors:** Ulysse Lebrec, Shambhu Sharma, Phil Watson, Rosine Riera, Hackmet Joer, Ryan Beemer, Christophe Gaudin

**Affiliations:** 1 Centre for Energy and Climate Geoscience, School of Earth Sciences, The University of Western Australia, Crawley, WA 6009 Australia; 2 Norwegian Geotechnical Institute, 40 St Georges Terrace, Perth, WA 6000 Australia; 3 Oceans Graduate School, The University of Western Australia, Crawley, WA 6009 Australia; 4 GTI Perth, Gnangara, WA 6077 Australia; 5 Department of Civil and Environmental Engineering, University of Massachusetts Dartmouth, Dartmouth, MA 02747 USA; 6 Oceans Institute, The University of Western Australia, Crawley, WA 6009 Australia

**Keywords:** Compressive strength, Carbonate sediments, Ooids, Calcarenite, North West Shelf, Cement, Civil engineering, Stratigraphy, Sedimentology, Geomorphology

## Abstract

Carbonate sediments are often regarded as problematic in geotechnical engineering due to the high variability of their properties. Understanding and quantifying this variability will become increasingly critical in the years ahead, notably with respect to upcoming developments in offshore renewable energy, for which limited in-situ data are typically available to characterise large areas. Here, six intervals from the North West Shelf of Australia, each composed of similar carbonate grains but accumulated in different environments, are investigated to better understand how the post-depositional cementation, alteration and dissolution of sediments, known as diagenesis, impact their geotechnical properties. Intervals are primarily affected by mineralogy-driven meteoric diagenesis, comprising in-situ dissolution of metastable grains and subsequent precipitation of cement that occurred when the shelf was exposed during lower sea-levels, and by marine diagenesis. In both cases, increased diagenesis results in a higher cement-to-solid ratio and compressive strength. However, while marine diagenesis is associated with a reduction in void ratio, this is not initially observed with mineralogy-driven meteoric diagenesis. Additionally, for a similar cement-to-solid ratio, microcrystalline cement results in higher compressive strength than sparite cement. The data further reveal that the level of meteoric cementation and the compressive strength increase as a function of the duration of exposure and of the regional climate, along with a reduction of the specific gravity related to the replacement of aragonite by calcite. However, increased meteoric diagenesis also leads to the formation of macro-scale heterogeneities such as calcrete layers and karsts that can affect the holistic geotechnical behaviour of such deposits.

## Introduction

The world’s continental shelves support and host a wide range of key ecological, renewable and hydrocarbon resources, and have been extensively surveyed and developed^1^. The recovery of both renewable and non-renewable energy resources commonly requires extensive offshore infrastructure and, in order to efficiently and safely design these facilities, knowledge of the seabed sediment and rock properties is essential^2^. A poorly defined ground model (defined as a combination of stratigraphical and geotechnical units interpreted from the geophysical and geotechnical data) or an incorrect estimation of the associated geotechnical properties may lead either to foundation performance challenges e.g. when driven piles were observed to free-fall through carbonate sediments during installation^3–5^ or in excessive design conservatism that has a direct (and typically negative) cost impact.

Carbonate sediments are present on many modern continental shelves^6^. When encountered, they are often described as being problematic for geoengineering purposes since their engineering properties often lie outside the ranges defined in the literature based on clay or silica sand^7^, for which design methodologies have largely been formulated. The different behaviour of carbonate deposits is generally associated with differences in grain property and genesis compared to siliciclastic sediments. While the accumulation of siliciclastic sediments is primarily related to the weathering of pre-existing rocks, and the mechanical transport and accumulation of the resulting grains^8^, the formation of carbonate sediments is primarily controlled by biological, chemical and physical processes^9^. Once deposited, the particles forming carbonate sediments may undergo several stages of transformation, which can lead to alteration, cementation and/or dissolution of the grains. These transformations, known as diagenesis^10^, are related to the chemistry of the surrounding fluid (e.g., fresh water/ brine water), changes in sea-level, emersion events or bacterial activity^11–13^. These processes lead to highly variable properties in terms of grain size, grain morphology, grain mineralogy, internal grain structure, intra- or inter-granular porosity and cementation. Moreover, diagenesis can occur to varying degrees, with Quaternary sediments often remaining in intermediate states of cementation, further complicating their assessment^14^.

Given the above discussion, it is therefore expected that carbonate units with the same origin, and which share the same carbonate content, grain size distribution, moisture content and porosity, can still exhibit radically different strength values^15^, making prediction of engineering properties challenging. In this regard, it has long been suggested that new classifications are required for carbonate material^15–17^.

Over the last few years, researchers have started to investigate how the nature of carbonate grains, and more specifically their internal structure, can affect geotechnical properties^18–20^. While these studies have improved understanding of the variability of carbonate sediment properties, they do not account for naturally occurring diagenetic transformations which can dramatically change the geotechnical properties of a sediment, and which have so far been largely understudied.

The North West Shelf (NWS) of Australia comprises one of the largest carbonate-dominated continental shelves formed in modern geological time^21^. The shelf is Australia’s main hydrocarbon province^22^ and hosts numerous offshore installations. The area has been extensively surveyed, including more than 350,000 km^2^ of 3D seismic surveys and countless exploration and geotechnical boreholes^23,24^. Further, the seabed is characterised by the presence of sedimentary features formed under both marine and meteoric environments^25^, making it the perfect ‘laboratory’ in which to study the impact of diagenesis on the geotechnical properties of carbonate sediments.

**Figure 1:**
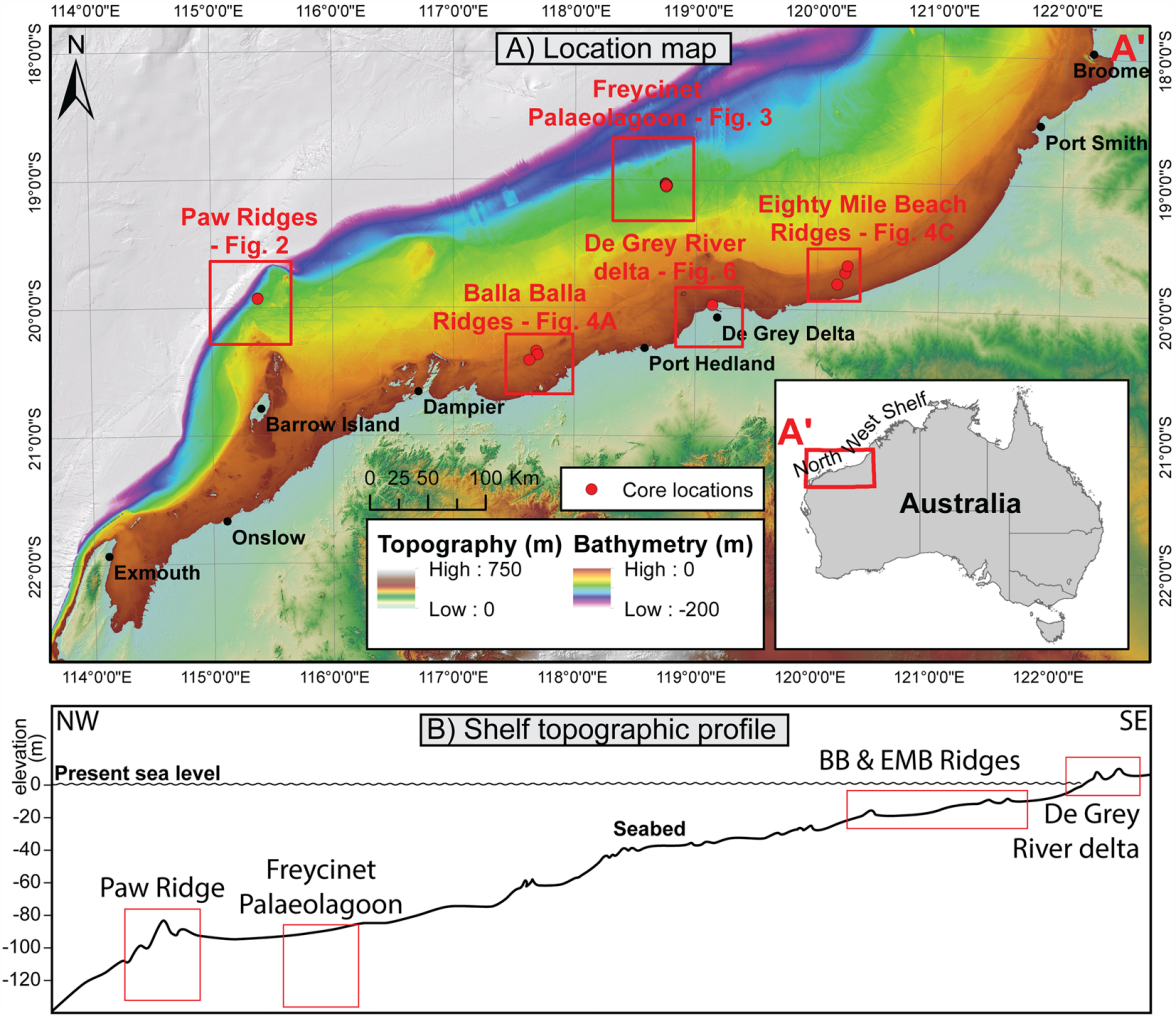
Study area—(**A**) Bathymetry of the area of interest; (**B**) Idealised cross-shelf topographic profile; Background digital elevation model from Lebrec et al.^24^; *BB* Balla Balla, *EMB* Eighty Mile Beach; Digital elevation models courtesy of Lebrec et al.^24^.

In this study, we investigate variably cemented sediments from five sites scattered along the NWS (Fig. 1) that were formed under varying diagenetic environments. The stratigraphy from each site was assessed using the framework of Lebrec et al.^26^ to identify intervals composed of similar grains, such that any change in geotechnical behaviour could be related purely to diagenesis. Following a laboratory testing program that included compressive strength testing, classification testing and petrographic measurements, the results from six discrete intervals were retained, with a particular focus on carbonate non-skeletal grains (NSGs), including both ooids and pelloids, due to their high homogeneity across sedimentary units. They are used in this paper to evaluate (1) how the nature of diagenesis impacts the geotechnical properties of the sediment; (2) the factors affecting the extent of diagenesis; and (3) how this knowledge can be used to reduce uncertainty and support future design.

## Regional setting

The NWS is a tropical passive margin extending over more than 2400 km along northwest Australia (Fig. 1) and is bounded by Exmouth and the Melville Islands^22,27^. The continental shelf itself is a gentle bathymetric ramp with regional slopes of less than one degree^27^. It can be divided into three depth zones based on the maximum depth of fair-weather and storm-weather wave action, as follows: (1) the ‘inner shelf’ from 0 to 50 m below sea level (bsl), (2) the ‘mid shelf’ from 50 to 120 m bsl and (3) the ‘outer shelf’ from 120 m bsl to the shelf break at roughly 200 m bsl^21,27,28^.

The shelf is characterised by the presence of numerous relict coastal features that were deposited during periods of lower sea level in response to wave, fluvial, tidal and aeolian processes^25,29,30^. These features consist mainly of cemented beach ridges, including barriers, strandplains and coastal dunes, which can be observed down to 180 m below sea level^25,31^. Locally, such relict sedimentary features support the development of both mesophotic and euphotic coral reefs^32–34^ and constitute biodiversity hotspots^35^. Across the shelf, the combination of tidal and oceanic currents has led to the formation of extensive fields of mobile bedforms^36–38^. Numerous pockmark fields are also observed at varying water depths along the shelf, attributed either to fluid seepage^39^, macro-tidal currents^40^ or biological activities^41^.

Overall, the NWS seabed sediments are largely dominated by palimpsest non-skeletal and skeletal carbonate grains and often have a carbonate content in excess of 90%^7,27^. Their distribution and subsequent cementation are controlled by the evolution of the climate, in relation to relative sea level^42^. Variably cemented Non-Skeletal carbonate Grains (NSGs), which are the focus of this study, consist of pelloids (typically aragonite-rich faecal pellets) and ooids (aragonite-coated grains developed around a nucleus) bound together by calcite cements^26,38^. NSGs were identified throughout the shelf, from the modern coast^26,38,43,44^ to the outer shelf^27,38,45^ indicating that they are a key contributor to the NWS sediments and are therefore of critical geotechnical interest.

## Datasets and methodology

### Data available and laboratory analyses

This study is based on the geotechnical laboratory testing of cores from five distinct site investigations conducted along the NWS of Australia between 2010 and 2021. The associated sites are respectively designated as Paw Ridges, Freycinet Palaeolagoon, Balla Balla Ridges, Eighty Mile Beach Ridges and De Grey River delta (Fig. 1) and were chosen to provide the best coverage of the seabed features likely to contain variably cemented NSGs in the cross-shelf direction.

The Paw dataset consists of five geotechnical cores taken from depths up to 75 m below seabed (bsb), that were collected as part of an offshore development using a piggyback coring system. All locations are within 50 m from each other and can be regarded as one composite location^26^. A total of 36 Uniaxial Compressive Strength (UCS) tests were conducted at the University of Western Australia using a triaxial cell with a constant vertical displacement rate of 1 mm/min. All samples had a dimension of 165 mm length × 62.5 mm diameter and were fully saturated. Testing also included measurement of the initial and final moisture content, as well as the dry density. In addition, classification testing included 35 carbonate content, dry density and specific gravity tests, 17 particle size distribution tests (conducted on the least-cemented samples) and 31 moisture content tests. These tests were conducted following the National Association of Testing Authorities (NATA) requirements.

The Freycinet geotechnical dataset consists of three geotechnical cores to a maximum depth of 48 m bsb, which were acquired using a remotely operated drill. Laboratory testing was undertaken at GTI Perth and included 13 UCS tests performed following ASTM D2166/D2166M-16^46^ as well as three Unconfined Direct Shear Stress tests, following the GTI-ADT-P1 proprietary method, that were used to derive an equivalent UCS value. Classification testing included 65 measurements of the carbonate content, dry density, specific gravity and moisture content following test method standards WA915.1 of the Main Roads Authority in Western Australia^47^, AS 1289 2.1.1^48^, and AS1289.3.5.1^49^ respectively. Particle size distribution tests were performed using AS 1289 3.6.1^50^ and AS 1289 3.6.3^51^.

The Balla Balla and Eighty Mile Beach datasets consist of six seabed cores of 3 m each, that were collected using a subsea drill rig operated by divers. Laboratory testing was performed at GTI Perth and included seven UCS (125 + mm length × 61 mm diameter), carbonate content and specific gravity tests performed following ASTM D2166/D2166M-16^46^, WA915.1^47^ and AS1289.3.5.1^49^ respectively.

Finally, The Centre for Energy and Climate Geoscience (UWA) conducted a field campaign along the De Grey River delta during which two samples were collected from outcropping cemented beach ridges. One sample (101.3 mm length × 53.9 mm diameter) was selected for laboratory testing and underwent a comparable testing program as the Balla Balla and Eighty Mile Beach samples. The particle size distributions of 18 adjacent uncemented subsamples were determined using laser scattering measurements.

The bathymetry used to provide geomorphological constraints was sourced from Lebrec et al.^24^ and supplemented by site-specific proprietary surveys where available and was displayed using ArcGIS Pro version 3.2. Lastly, 140 thin sections sourced from Lebrec et al.^26^ and stained with alizarin red S and potassium ferricyanide were analysed to further characterise the nature of the grains and cements. Cement-to-solid ratios (solid encompasses both grains and cements) were visually estimated using visual aid charts from Terry and Chilingar^52^ and Stevenson^53^.

### Data interpretation

Petrographic grain and cement descriptions build on the methods and nomenclatures from Scholle and Ulmer-Scholle^13^ and Gallagher et al.^54^, while geotechnical classifications follow Clark and Walker^55^, which is widely used in the offshore geotechnical industry. It should be noted that the term ‘microcrystalline cement’ was preferred over ‘micrite’ to refer to cement crystals with a diameter of less than 20 μm to avoid confusion between depositional ooze and cement, following the definition from Friedman^56^ and Dunham and Ham^57^. In contrast, the term ‘sparite’ is used to refer to larger cement crystals.

The calculation of void ratio, porosity and saturation using measured specific gravity, dry density and moisture content was performed using the equations from Craig^58^. In the case of UCS test results for which specific gravity values were not always available, values were selected from the closest classification test location (within the same sedimentary unit).

UCS tests available for this study were performed either on saturated samples (Paw, Freycinet) or air-dried samples (Balla Balla, Eighty Mile Beach, De Grey). Given that increased water saturation has a strong decreasing effect on UCS peak stress^59–61^, a weakening factor has been adopted in this paper to allow comparison of saturated and unsaturated test results. Since this factor could not be defined empirically (due to a lack of overlapping data) it was set to 0.5—which corresponds to the average weakening factor of similar sediments presenting equivalent levels of cementation published by Price^62^, Chang-qi et al.^63^, Ciantia et al.^64^, Kasim and Shakoor^65^, Rabat et al.^66^, Rabat et al.^67^, Rashed et al.^68^ and Vásárhelyi^69^.

## Results

As introduced previously, six variably cemented intervals containing more than 80% of NSGs (such as ooids and peloids) were identified from five areas of interest. Each interval and the associated geotechnical properties are described hereafter.

### Paw ridge

**Figure 2:**
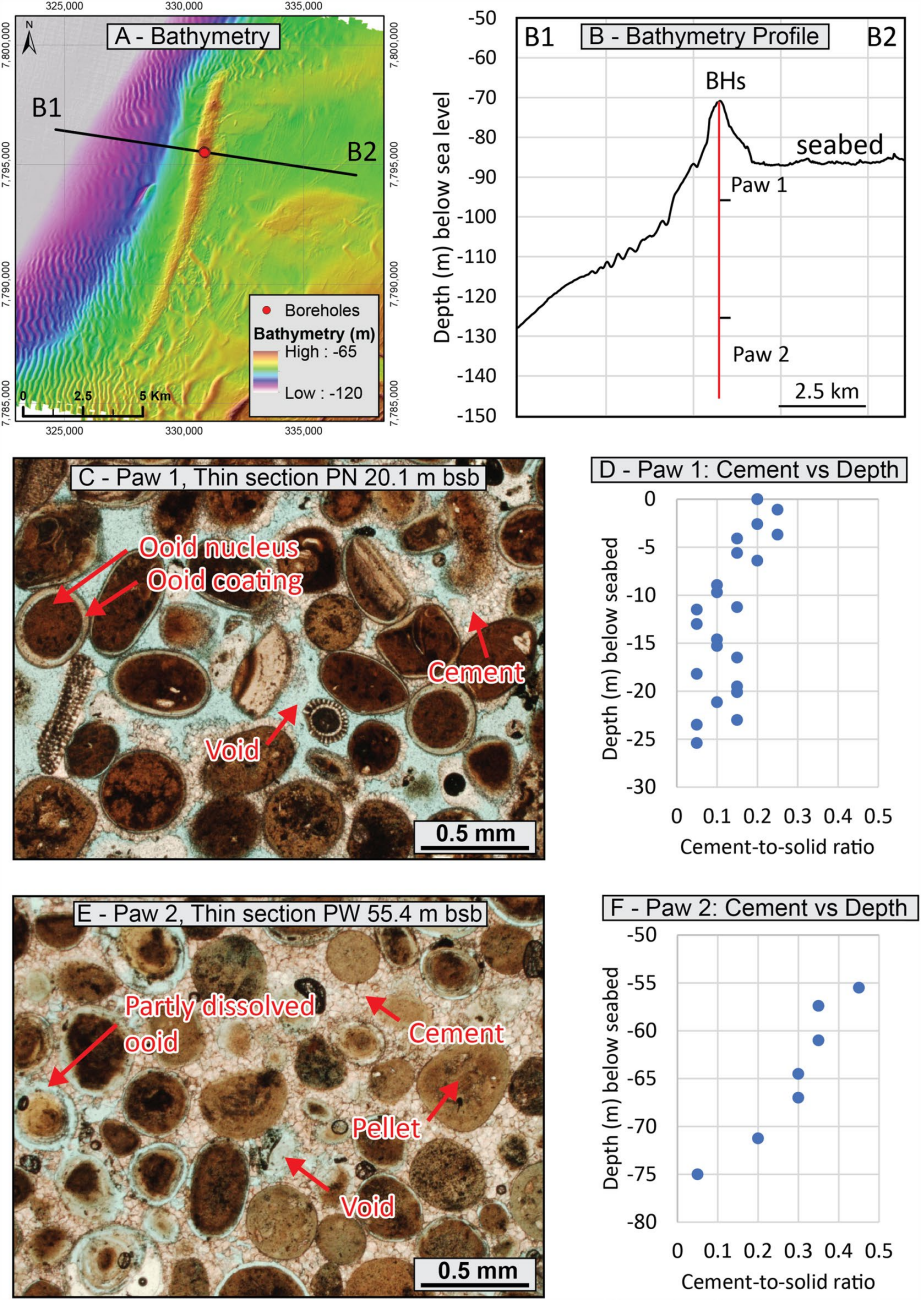
Paw Ridge sedimentary units—(**A**) Seabed morphology and borehole locations; (**B**) Bathymetry profile; (**C**) Paw 1 (uppermost interval) sedimentary microfacies—note variable cement infill; (**D**) Paw 1 evolution of the cement-to-solid ratio with depth; (**E**) Paw 2 (lowermost interval) sedimentary microfacies; (**F**) Paw 2 evolution of the cement-to-solid ratio with depth; Digital elevation model courtesy of the Centre for Energy and Climate Geoscience industry partners.

The Paw Ridge is located in 100 m water depth and rises to around 32 m above the surrounding seabed (Fig. 2A,B). The ridge has an actual height in excess of 75 m but is largely buried by younger sediments. It consists of three superimposed smaller ridges that were previously interpreted as a succession of relict aeolianites and coral reefs deposited during lower sea levels^26^. NSG-rich grainstones were identified within the lowermost and uppermost ridges.

The Paw 1 interval corresponds to the uppermost ridge and extends from the seabed to a depth of about 25 m bsb. The interval, previously interpreted as an aeolianite accumulated during Marine Isotopic Stage 2, is composed of very well sorted NSGs that exhibit local bedding inclined by up to 34 degrees^26^. The interval is variably cemented both at the macro and micro scale, with cemented zones developed adjacent to loose sediment (Fig. 2C). The cement consists of sparite crystals which variably fill pore space and locally form menisci for an average cement-to-solid ratio of 0.135 which decreases with depth (Table 1, Fig. 2D). A total of 20 UCS and 23 classification tests were conducted within the Paw 1 interval. It is characterised by an average carbonate content of 99.5% which is associated with an average specific gravity of 2.85 (Table 2). The sediment has an average water content of 19.76% and an average dry density of 1.79 g/cm^3^, which translates into an average void ratio, porosity and saturation of 0.59, 37.2% and 95.7%, respectively. The sediment has a mean particle size (D_50_) of 0.223 mm which classifies it as sand. UCS peak stress values range from around 50 to over 1500 kPa (Table 3) which, given that the grains are homogeneous throughout, illustrates varying degrees of diagenesis. Based on the above, the sedimentary unit ranges from carbonate sand to calcarenite following the Clark and Walker^55^ classification.

**Table 1:** Cement-to-solid ratio of the NSGs-rich intervals.

Unit	Cement-to-solid ratio			
	n*	Avg	Min	Max
Paw1	24	0.135	0.05	0.25
Paw2	7	0.286	0.05	0.45
Freycinet	7	0.082	0.025	0.15
Balla Balla	12	0.437	0.2	0.75
Eighty Mile Beach	6	0.292	0.05	0.5
De Grey	*2*	0.10	0.10	0.10

*Number of thin sections described.

**Table 2:** Classification properties of the NSG-rich intervals.

Unit	BH	From (m)	To (m)	CaCO_3_ (%)	G_s_ (g/cm^3^)	ρ_d_ (g/cm^3^)	w (%)	e	Øt (%)	S_r_ (%)	D_50_ (mm)	Cement
Paw1	P-S	1.1	1.4	99.7	2.84	1.75	21.8	0.62	38.4	99.4	0.239	Sparite
Paw1	P-S	4.0	4.3	99.5	2.85	1.79	20.8	0.592	37.2	100.1	0.241	Sparite
Paw1	P-S	7.2	7.5	98.8	2.85	1.84	18.7	0.55	35.4	97.1	0.227	Sparite
Paw1	P-S	10.0	10.3	99.5	2.85	1.85	18.6	0.541	35.1	98.1	0.227	Sparite
Paw1	P-S	17.2	17.5	99.6	2.87	1.78	20	0.612	38.0	93.7	0.196	Sparite
Paw1	P-S	23.5	23.8	99.3	2.85	1.81	19.3	0.575	36.5	95.7	0.208	Sparite
Paw1	P-N	1.2	1.4	99.4	2.86	1.75	22	0.634	38.8	99.2	0.222	Sparite
Paw1	P-N	6.8	7.0	99.6	2.85	1.87	17.9	0.524	34.4	97.3	0.247	Sparite
Paw1	P-N	10.3	10.6	99.5	2.88	1.78	20.3	0.618	38.2	94.6	0.234	Sparite
Paw1	P-N	14	14.3	98.7	2.85	1.78	22.7	0.601	38.0	107.6	0.234	Sparite
Paw1	P-N	20.7	21	98.7	2.86	1.85	19.8	0.550	35.0	103.7	0.219	Sparite
Paw1	P-E	0.33	0.45	99.0	2.85	–	–	–	–	–	–	Sparite
Paw1	P-E	5.4	5.46	99.8	2.85	–	–	–	–	–	–	Sparite
Paw1	P-E	8.5	8.6	99.9	2.85	–	–	–	–	–	–	Sparite
Paw1	P-E	10.3	10.3	99.7	2.85	–	–	–	–	–	–	Sparite
Paw1	P-E	14.4	14.5	99.9	2.85	–	–	–	–	–	–	Sparite
Paw1	P-E	18.3	18.5	99.9	2.85	–	–	–	–	–	–	Sparite
Paw1	P-E	23.6	23.8	98.7	2.82	–	–	–	–	–	0.119	Sparite
Paw1	P-W	1.1	1.45	99.8	2.85	–	–	–	–	–	0.256	Sparite
Paw1	P-W	4.5	4.8	99.5	2.85	–	–	–	–	–	0.216	Sparite
Paw1	P-W	9	9.3	99.7	2.87	1.84	18.5	0.560	36.0	94.9	0.223	Sparite
Paw1	P-W	17.25	17.5	99.7	2.86	1.85	17.8	0.546	35.3	93.24	0.225	Sparite
Paw1	P-W	23.2	23.5	99.7	2.8	1.56	18.5	0.790	44.0	65.2	0.252	Sparite
Average				99.5	2.85	1.79	19.76	0.594	37.2	95.7	0.223	–
Paw2	P-W	54.4	54.5	99.7	2.84	1.76	20.7	0.614	38.0	95.8	–	Sparite
Paw2	P-W	63.3	63.5	99.8	2.83	1.69	23.8	0.675	40.3	99.9	–	Sparite
Paw2	P-W	69.6	69.7	99.6	2.84	1.51	30.7	0.880	46.8	99.0	–	Sparite
Paw2	P-W	74.1	74.2	99.6	2.87	1.8	23.4	0.594	37.3	112.3	–	Sparite
Average				99.7	2.85	1.69	24.7	0.691	40.6	101.9	–	–
Freycinet	BH03	36.0	36.2	98.1	2.83	1.56	27.2	0.810	44.9	94.6	–	MCC
Freycinet	BH03	36.8	36.9	–	–	–	–	–	–	–	0.189	MCC
Freycinet	BH02	38.5	38.7	97.5	2.77	1.4	37.2	0.979	49.5	105.3	0.261	MCC
Freycinet	BH01	35.1	35.3	98.7	2.76	1.40	28.2	0.971	49.3	80.1	–	MCC
Freycinet	BH04	34.9	35.1	99.1	2.81	1.72	20.6	0.634	38.8	91.3	–	MCC
Average				98.4	2.79	1.52	28.3	0.849	45.6	92.8	0.225	–
BB	BB4	1.6	1.7	98.1	2.81	1.79	4.9	0.570	36.3	24.2	–	Sparite
BB	BB5	2.9	3.0	93.2	2.72	–	–	–	–	–	–	Sparite
EMB	EMB3	2.1	2.2	98.7	2.75	1.65	3.9	0.667	40.0	16.1	–	Sparite
EMB	EMB4	1.1	1.2	98.9	2.80	1.59	4.3	0.761	43.2	15.8	–	Sparite
Average				97.2	2.77	1.68	4.4	0.67	39.8	18.7	–	–
DeGrey	DG16	0	0.1	61.7	2.81	1.82	8.1	0.540	35.2	41.8	0.502	Sparite

*G*_*s*_ specific gravity, *ρ*_*d*_ dry density, *w* water content, *e* void ratio, *Øt* total porosity, *S*_*r*_ degree of saturation—values > 100 may result from density measurement uncertainties, *D*_*50*_ median particle size, *MCC* microcrystalline calcite.

**Table 3:** UCS test results of NSG-rich intervals.

Unit	BH	From (m)	To (m)	w (%)	ρd (g/cm^3^)	σUCS (kPa)	ε_a_ (%)	E (MPa)	e	Øt (%)	Sr (%)	Cement
Paw1	P-S	4.45	4.62	18.0	1.83	72.1	2.2	13	0.56	35.8	92.0	Sparite
Paw1	P-S	10.80	11.00	16.2	1.86	109.8	1.0	16	0.53	34.7	86.7	Sparite
Paw1	P-S	13.40	13.51	17.3	1.85	125.2	1.2	13	0.54	35.1	91.2	Sparite
Paw1	P-S	17.00	17.20	19.2	1.81	53.5	0.7	29	0.59	36.9	94.1	Sparite
Paw1	P-S	19.00	19.30	19.3	1.85	139.0	1.7	11	0.55	35.5	100.0	Sparite
Paw 1	P-S	23.90	24.02	15.0	1.90	99.9	0.7	19	0.50	33.3	85.5	Sparite
Paw1	P-N	0.15	1.00	21.1	1.76	428.7	0.9	71	0.63	38.5	96.6	Sparite
Paw1	P-N	6.32	6.48	21.6	1.79	247.1	2.3	14	0.59	37.2	104	Sparite
Paw1	P-N	14.40	14.60	20.8	1.77	308.2	1.1	46	0.61	37.9	97.2	Sparite
Paw1	P-N	22.2	22.35	16.4	1.91	162.4	1.1	23	0.50	33.2	94.3	Sparite
Paw1	P-N	25	25.17	19	1.81	373.9	1.5	47	0.547	35.4	97.2	Sparite
Paw1	P-E	3.46	3.63	19.3	1.76	116.5	2.2	12	0.62	38.2	88.8	Sparite
Paw1	P-E	6.70	6.87	20.8	1.75	882.5	1.2	126	0.63	38.6	94.3	Sparite
Paw1	P-E	10.00	10.25	18.9	1.71	718.3	0.9	128	0.67	40.0	80.8	Sparite
Paw1	P-E	14.50	14.70	15.9	1.91	583.1	0.9	99	0.49	33.0	92.1	Sparite
Paw1	P-E	17.80	17.98	11.5	1.97	1541.9	1.1	316	0.45	30.9	73.4	Sparite
Paw1	P-E	23.20	23.32	12.8	1.88	91.9	0.5	17	0.50	33.3	72.2	Sparite
Paw1	P-W	12.10	12.30	18.1	1.83	94.1	1.1	11	0.57	36.2	91.4	Sparite
Paw1	P-W	21.40	21.56	16.7	1.83	63.4	1.8	5	0.53	34.6	88.2	Sparite
Paw1	P-W	22.80	22.92	17.4	1.86	121.1	1.2	15	0.51	33.6	96.4	Sparite
Average				17.8	1.83	316.6	1.25	51.6	0.56	35.6	90.7	–
Paw2	P-W	54.20	54.37	21.2	1.75	2839.4	0.9	562	0.62	38.4	96.7	Sparite
Paw2	P-W	59.20	59.34	21.2	1.71	748.1	2.0	66	0.65	39.6	91.6	Sparite
Paw2	P-W	63.50	63.66	21.1	1.72	1306.4	1.6	153	0.65	39.2	92.5	Sparite
Paw2	P-W	69.30	69.41	28.0	1.53	275.0	0.6	64	0.86	46.1	92.9	Sparite
Average				22.9	1.68	1292.2	1.3	211	0.70	40.8	93.4	–
Freycinet	BH01	36.80	37.01	34.5	1.41	632.0	0.64	226	0.96	48.9	99.5	MCC
Freycinet	BH01	30.8	31.03	35.6	1.12	95.6	–	–	1.46	59.4	67.1	MCC
Freycinet	BH04	32.9	33.07	18.7	1.78	4473.0	0.7	1071	0.58	36.7	90.8	MCC
Freycinet	BH04	35.1	35.36	21.4	1.66	2144.0	0.2	2000	0.69	40.9	86.8	MCC
Freycinet	BH03	33.7	33.86	26.8	1.52	1524.0	0.2	889	0.86	46.3	88.0	MCC
Freycinet	BH03	36.2	36.37	24.0	1.61	1869.0	0.3	1000	0.76	43.1	89.6	MCC
Average				26.8	1.52	1789.6	0.4	1037	0.89	45.9	87.0	–
BB	BB4	1.60	1.73	4.9	1.79	2640.0	0.4	723	0.57	36.3	24.2	Sparite
BB	BB5	2.90	3.04	–	1.7*	8723.0	–	–	0.6*	37.5*	–	Sparite
EMB	EMB3	2.10	2.24	3.9	1.65	8250.0	1.3	783	0.67	40.0	16.1	Sparite
EMB	EMB4	1.10	1.23	4.3	1.59	3940.0	0.7	758	0.76	43.2	15.8	Sparite
Average				4.37	1.68	5888.0	0.8	755	0.65	39.25	18.7	–
DeGrey	DG16	0.00	0.10	8.1	1.82	1814.0	0.2	645	0.54	35.2	41.8	Sparite

*w* water content, *ρd* dry density, *σUCS* peak stress, *ε*_*a*_ axial strain at peak stress, *E* Young’s modulus, *e* void ratio, *Øt* total porosity, *Sr* degree of saturation, *MCC* microcrystalline calcite. Note that the void ratio, total porosity and degree of saturation were calculated using the specific gravity from the closest classification test, within the same unit; *value derived from a visual estimate of the void ratio.

The Paw 2 interval corresponds to the lowermost ridge, extending from 50 m bsb to the end of the available borehole (at around 75 m bsb). This interval, previously interpreted as an older relict aeolianite formed during Marine Isotopic Stage 6^26^, has been described using four UCS and classification tests. It presents a facies that is very similar to Paw 1 and is characterised by the presence of very well sorted NSGs (Fig. 2E) that exhibit steep bedding, and is typically cemented by sparite cements. The primary difference between the two Paw intervals is in the level of cementation—Paw 2 (which is older) is more cemented than Paw 1, comprising an average cement-to-solid ratio of 0.286 (Table 1) and with grains that are often partly to fully dissolved. Additionally, the level of cementation and dissolution appears to increase toward the top of the interval (Fig. 2F). This is reflected in the UCS tests, which show an average peak stress of around 1300 kPa but a maximum value of more than 2800 kPa observed at the top of the interval (Table 3). In contrast, the classification properties are very similar to those of Paw 1—with average specific gravity, dry density, carbonate content and moisture content of 2.85, 1.69 g/cm^3^, 99.7% and 24.7%, respectively (Table 2). Similarly, the interval has a void ratio of 0.69, corresponding to a porosity of 40.6% and a saturation > 95%. The particle size distribution could only be assessed visually due to the induration of the interval, with a mean (D_50_) value estimated to be on the order of 0.25–0.5 mm (i.e. sand-size). The interval, given the level of cementation, is classified as calcarenite following Clark and Walker^55^.

### Freycinet palaeolagoon

**Figure 3:**
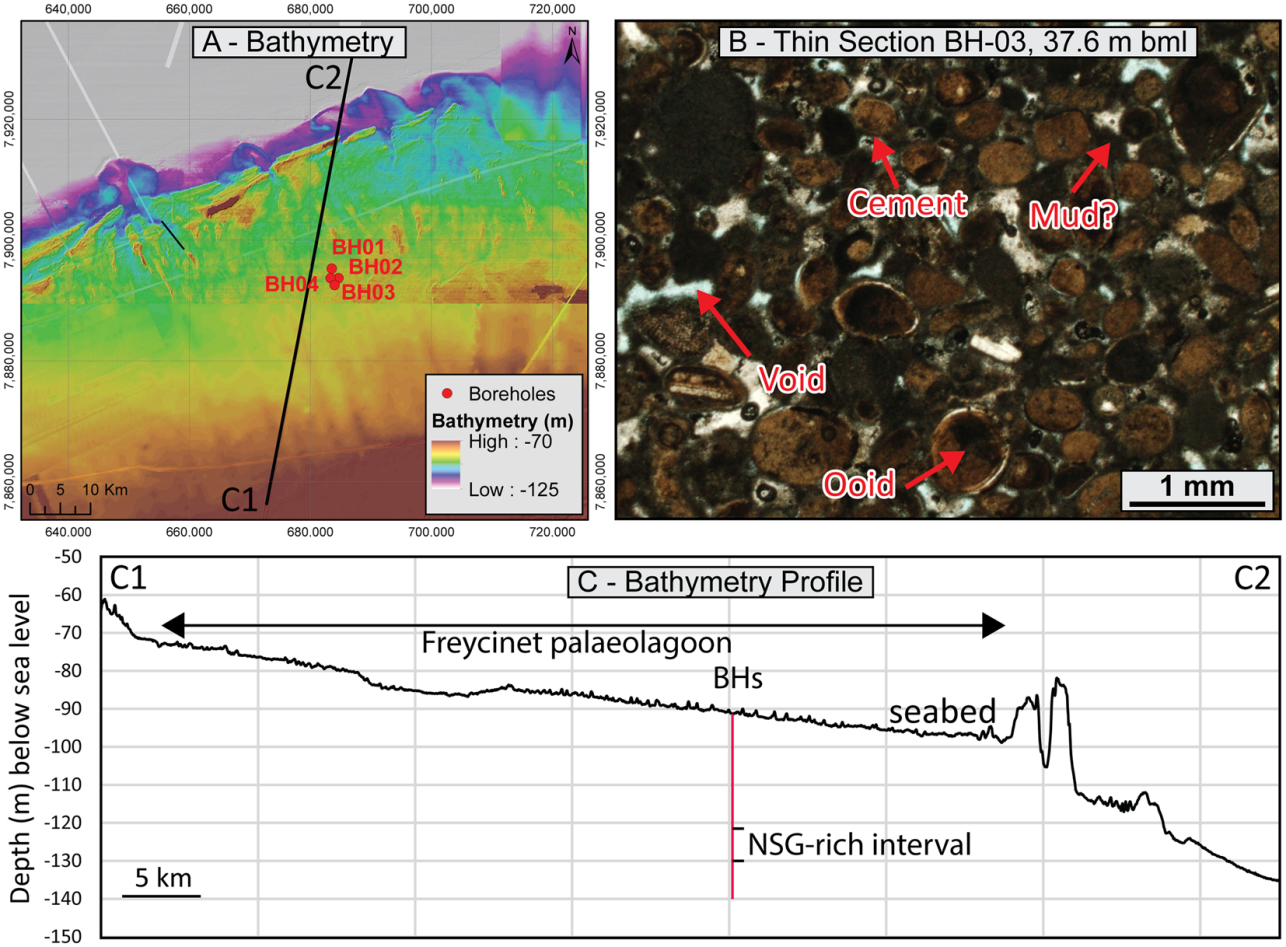
Freycinet sedimentary unit—(**A**) seabed morphology and borehole locations; (**B**) Sedimentary microfacies; (**C**) Bathymetry profile; Bathymetry produced using data courtesy of TGS and Lebrec et al.^24^.

The Freycinet Lagoon site lies in 89 m water depth, within a palaeolagoon that was formed during the last glacial period (Fig. 3A,C). The cores inspected as part of the current study are characterised by a succession of open shallow-water marine and protected lagoonal deposits^26^. NSGs were identified at depths ranging from 30 to 36 m bsb and interpreted to have accumulated within protected shallow-marine to sub-tidal shoals during marine isotopic stage 4. The cement comprises primarily microcrystalline crystals and the interval is typically well-lithified (Fig. 3B). Determination of the cement-to-solid ratio was difficult due to the mix of carbonate mud with microcrystalline cement, but is estimated to vary between 0.025 and 0.15 (Table 1). Grains are locally dissolved and may be partially filled with sparite crystals.

The geotechnical properties of the interval were investigated using six UCS and five classification tests. The samples have an average carbonate content of 98.4% and a specific gravity of 2.79 (Table 2). Dry density values are the lowest encountered in this study (at an average of 1.52 g/cm^3^) and translate to average void ratio and porosity of 0.85 and 45.6%, respectively. The interval has an average moisture content of 28.3%, and an average saturation of 92.8%. Particle size distribution analyses returned an average D_50_ of 0.225 mm. UCS peak stress values range from around 100 kPa to nearly 4500 kPa, for an average of around 1800 kPa (Table 3)—it should be noted that the lowest value is located at the very top of the unit, near the interface with the overlying interval, and may not be representative of the majority of the layer. The interval is classified as calcarenite following Clark and Walker^55^.

### Balla Balla and eighty mile beach ridges

**Figure 4:**
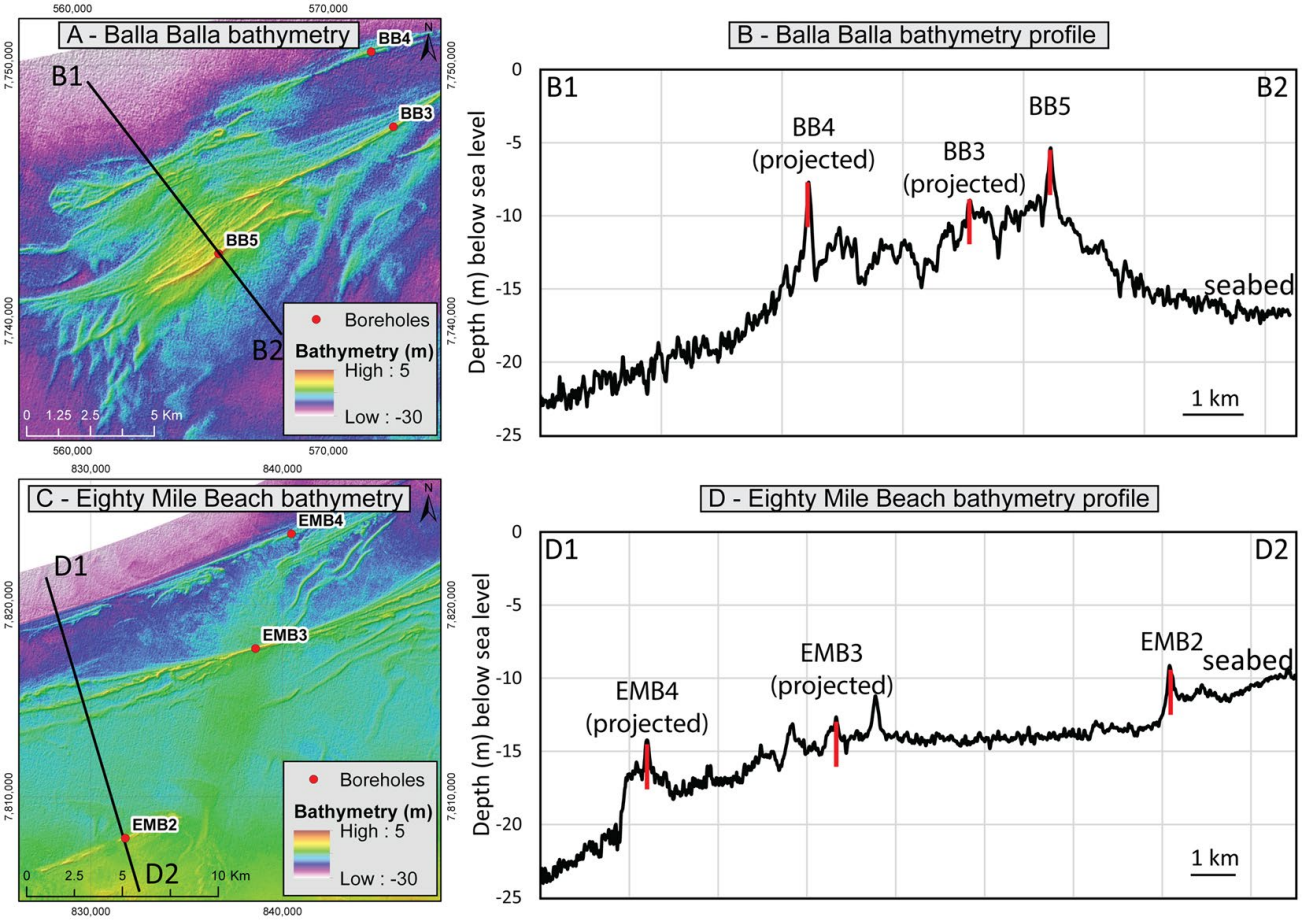
Inner shelf ridges—(**A**) Balla Balla seabed morphology and borehole locations; (**B**) Bathymetry profile; (**C**) Eighty Mile Beach seabed morphology and borehole locations; (**D**) Bathymetry profile; Digital elevation models courtesy of Lebrec et al.^24^.

The Balla Balla ridges are located 20 km offshore of Depuch Island in 7–15 m of water depth (Fig. 4A), whereas the Eighty Mile Beach ridges are located 15–20 km offshore Eighty Mile Beach and lie in 5–18 m water depth (Fig. 4C). Both series of ridges are up to 1000 m in width and 5 m in height (Fig. 4B,D), and are part of the same track of ridges that run parallel to the coast for over 1000 km. They were formed during Marine Isotope Stage 5 and were previously interpreted as the aeolian caps of relict regressive strandplains^26^.

**Figure 5:**
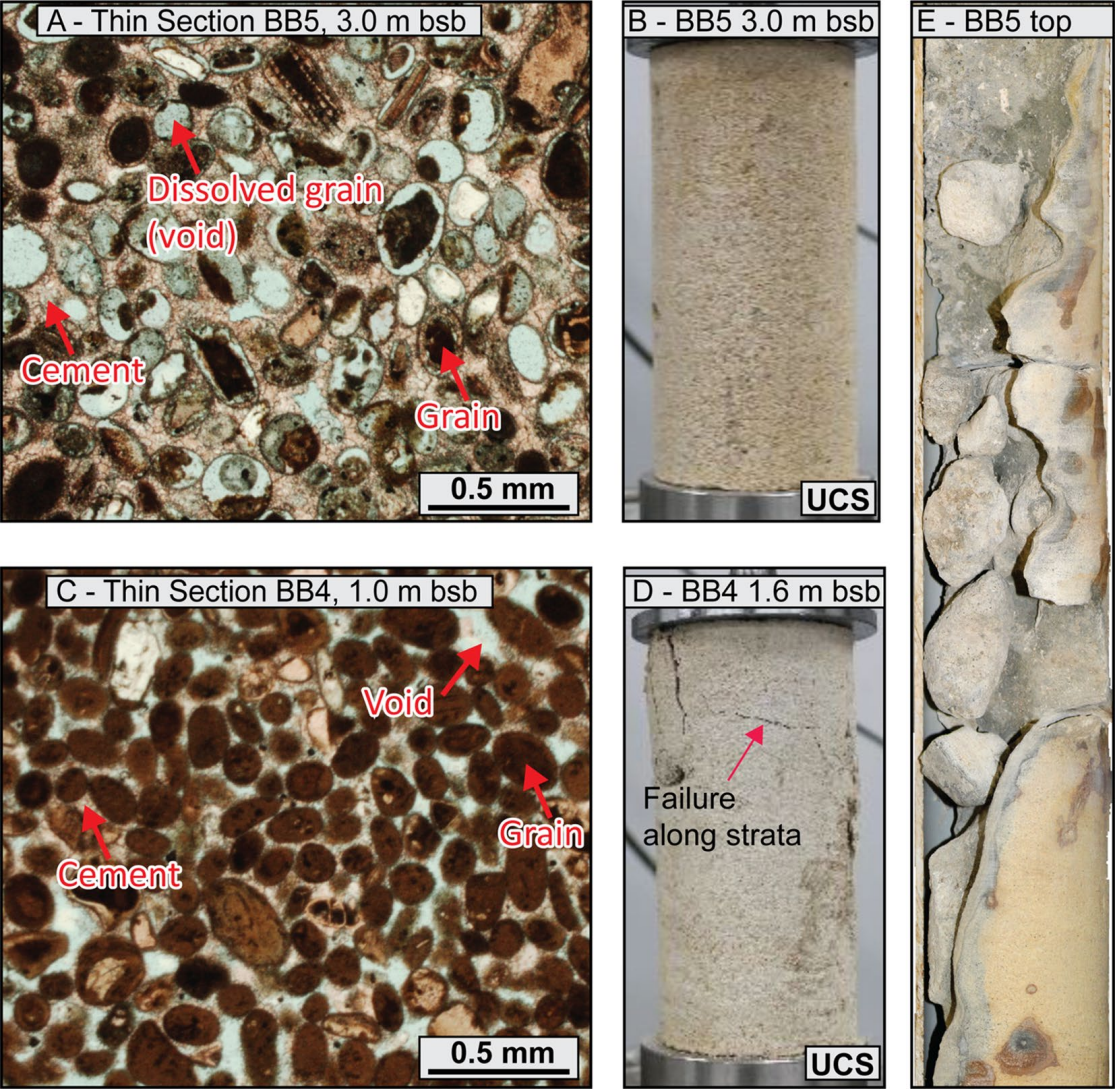
Balla Balla and Eighty Mile Beach Ridges sedimentary facies—(**A**) Moldic interval sedimentary microfacies (note the near-complete dissolution of the grains in light blue); (**B**) Moldic interval (BB3) reached UCS apparatus limit; (**C**) Partly cemented interval sedimentary microfacies; (**D**) In such interval failure occurred along less cemented strata (BB4); (**E**) Karstified interval (note the extent of dissolution); All cores have a diameter of 6.1 cm.

The ridges are primarily composed of well-sorted NSGs, although in some cases include rare bioclasts (Fig. 5A,C). They are partly to well cemented with sparite crystals, resulting in an average cement-to-solid ratio of 0.40. Some of the NSGs are fully dissolved, and in places the inversion of porosity is almost complete (Fig. 5A)—leading to a cement-to-solid ratio in excess of 0.7, in which case the interval is referred to as moldic (meaning the grains are fully dissolved, and the inter-granular pore space is now filled with cement). Locally, centimetric-to-metric dissolution pipes and vugs intersect the cores (Fig. 5E), which can be associated with karst breccia. Additionally, the top of the ridges often presents a centimetres-thick calcrete.

The properties of four samples (one per main ridge) were tested in the laboratory, noting that the calcrete layers could not be tested due to their limited thickness. The ridges present an average carbonate content of 97.2% and a specific gravity of 2.77 (Table 2). The average dry density of 1.68 g/cm^3^ translates to a void ratio of 0.67 and a porosity of 39.8%. It should be noted that the void ratio and porosity values of the moldic intervals appear slightly lower than those of the remaining material (by around 0.1 and 5%, respectively). A similar disparity is observed with UCS values—non-moldic intervals (e.g., Fig. 5C) show an average UCS of around 3300 kPa, while moldic intervals (e.g., Fig. 5A) have an average UCS of nearly 8500 kPa (Table 3). The latter is likely an underestimate given that one of the two tests conducted on moldic intervals reached the limit of the apparatus (Fig. 5B). On the other hand, the lowest UCS values appear to be associated with failure occurring along the strata, potentially illustrating weakened layers (Fig. 5D). In any case, the samples had an average moisture content of 4.4% and a degree of saturation of 18.7%—note these values do not represent in situ conditions, as such data was not available. The level of induration prevented an evaluation of the particle size distribution, although the D_50_ was visually estimated (from thin sections) as ranging between 0.25 and 0.5 mm, with the ridges therefore classified as calcarenite^55^.

### De Grey River delta

**Figure 6:**
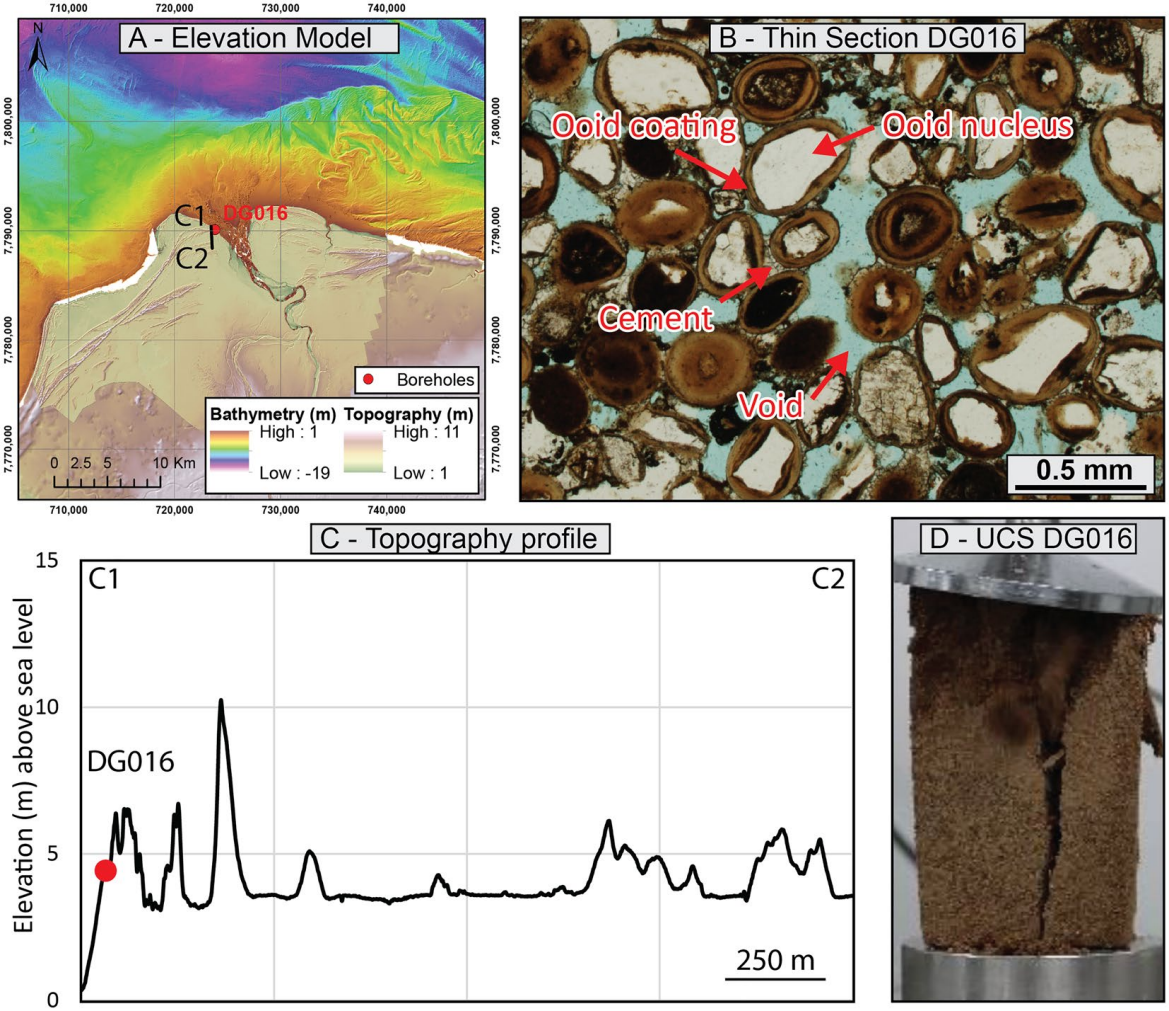
De Grey Delta coastal ridges—(**A**) onshore and offshore morphology and sample locations; (**B**) Sedimentary microfacies—note the quartz (white) within the ooids; (**C**) Topography profile; (**D**) UCS test failure; Digital elevation models courtesy of Lebrec et al.^24^ and Lebrec et al.^38^.

The De Grey River delta is the largest and most active delta of the North West Shelf^70^. Recent onshore and offshore site investigations reveal that the delta sedimentary features are composed predominantly of ooids, which have developed around terrestrial siliciclastic nuclei^38^. The outer rim of the delta is characterised by the presence of several late Holocene ooid-rich beach ridges (Fig. 6A,C), which appear uncemented but typically contain a lithified core that is visible through road cuts and trenches. The cement is comprised of sparite calcite crystals that form extensive bridges (menisci) between grains, resulting in a cement-to-solid ratio of 0.10 (Fig. 6B, Table 1).

Only one sample was investigated in the laboratory (Fig. 6D). This sample has a carbonate content of 61.7% (Table 2), which is much lower than the other intervals presented in this study—reflecting the terrigenous clastic origin of the ooid nuclei. The sample has a specific gravity of 2.81 and a dry density of 1.82 g/cm^3^, which translates into a void ratio of 0.54 and a porosity of 35.2%. The moisture content was measured at 8.1% and indicates that only 41.1% of the pores are saturated—again, this may not reflect in-situ conditions. The particle size distribution could not be determined on the cemented interval and was performed instead on adjacent (loose) sediment, returning a D_50_ of around 0.5 mm. With a UCS test resulting in a peak stress of around 1800 kPa (Table 3), the sample is categorised as calcarenite following Clark and Walker^55^.

## Discussion

### Influence of cement type

The six intervals investigated in this study comprise non-skeletal carbonate grains (ooids and peloids) with similar grain size and sorting, which are bound together by post-depositional cementation and underwent only limited compaction. As a result, changes in compressive strength and associated properties are thought to be primarily related to the type of cement and level of cementation. Two main types of cement can be identified, namely (1) sparite and (2) microcrystalline. The formation of these cements is associated with early marine and meteoric diagenesis and no evidence of burial or deformation diagenesis was found.

#### Sparite cement

**Figure 7:**
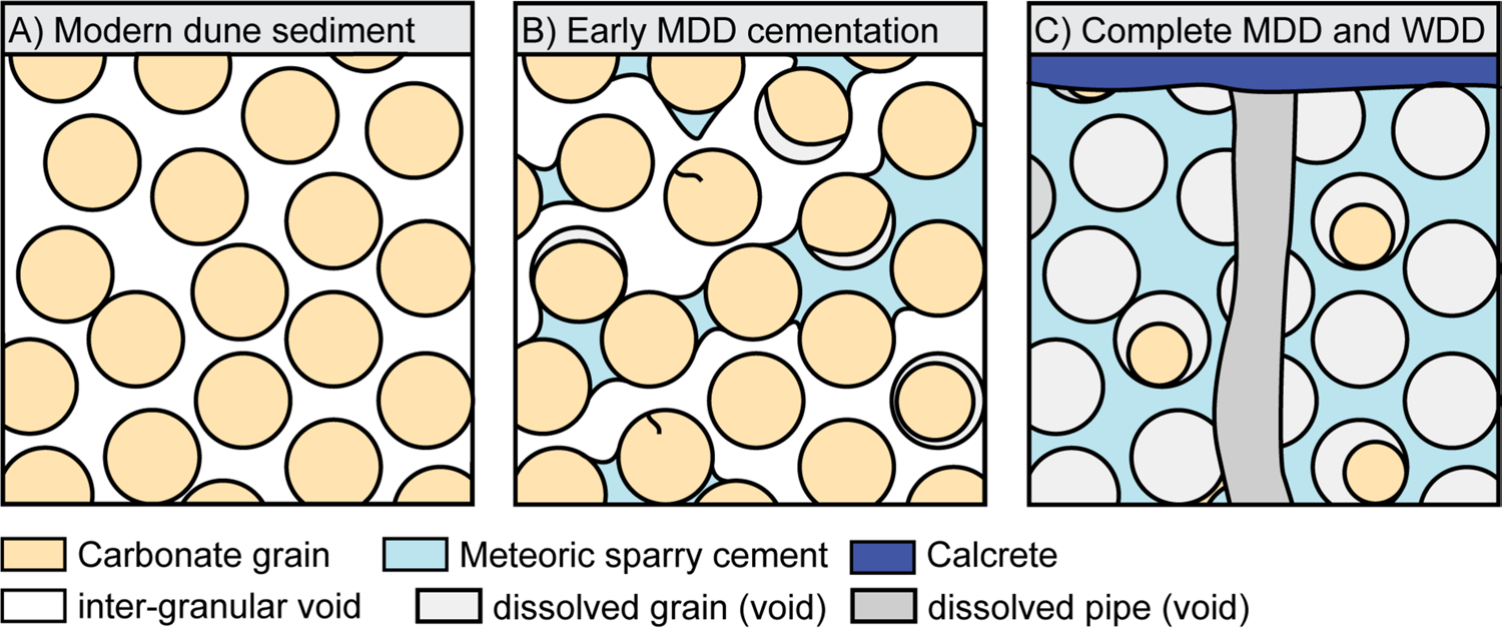
Summary sketch of the meteoritic diagenesis observed in this study—(**A**) Loose sediment; (**B**) Mineral-driven meteoric diagenesis (MDD) initially result from the dissolution of aragonite grains and the subsequent precipitation of calcite cement in the pore space; The cement variably fills pore space, illustrating the partial water saturation of the sediment, and cemented intervals can laterally transition to uncemented sediments; (**C**) The process ultimately leads to an inversion of the porosity where all grains are dissolved and inter-granular porosity is filled with cements; The circulation of water can lead to water-driven diagenesis (WDD) resulting in the formation of dissolutions features (karts) and calcrete.

Sparite cement was primarily identified within the Paw Ridge, Balla Balla Ridges, Eighty Mile Beach Ridges and De Grey River delta samples. The cement variably fills the pore space and locally forms menisci, reflecting the partial saturation of the sediment at the time of cementation (Fig. 7), in a pattern that is typical of meteoric cementation^9^.

**Figure 8:**
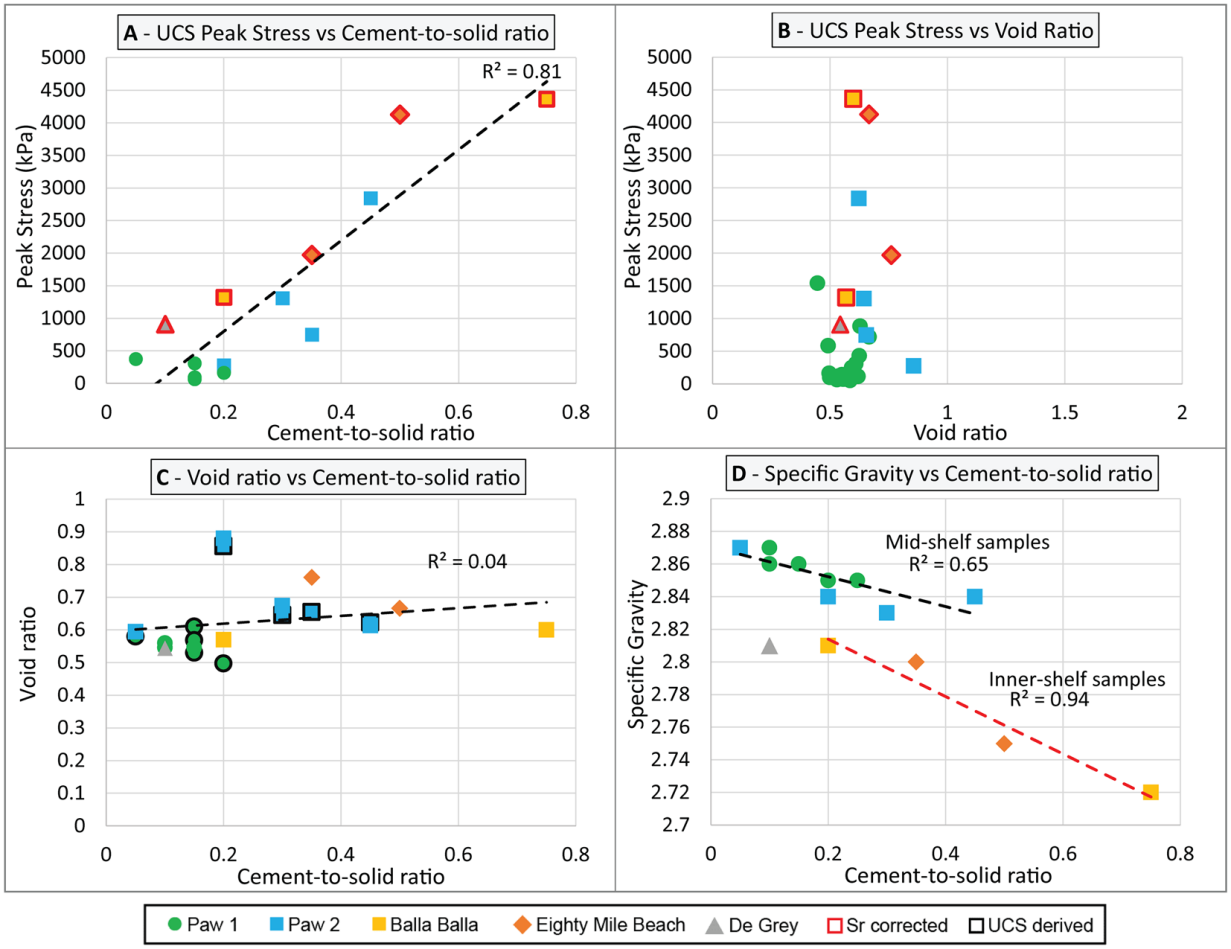
Properties of NSG-rich grainstone cemented as a result of mineral-driven meteoric diagenesis—(**A**) UCS Peak stress versus cement-to-solid ratio; (**B**) UCS Peak stress versus void ratio; (**C**) Void ratio versus cement-to-solid ratio; Black-contour labels indicate void ratio values that were calculated using dry density values from UCS tests; (**D**) Specific gravity versus cement-to-solid ratio; Sr corrected labels indicate datapoints that include a weakening factor of 0.5 to take into account variable water-saturation rates (see “Data interpretation” section for how this value was determined).

Overall, an increase in the degree of meteoric diagenesis, as illustrated by an increase in the cement-to-solid ratio (e.g., Fig. 7B versus Fig. 7C), is characterised by an increase in compressive strength (Fig. 8A). Scatter in the test data highlights uncertainties associated with visual estimates of cement-to-solid ratio, but also the micro-scale heterogeneities associated with meteoric diagenesis: localised areas or strata can remain either more dissolved or less cemented than others, resulting in the development of preferential failure planes. There is no clear relationship between void ratio, cement-to-solid ratio and peak UCS values (Fig. 8B,C, r^2^ = 0.04). This is thought to relate to the mechanisms driving cementation. In meteoric environments, initial cementation of the sediment results from the dissolution of metastable aragonitic and high-magnesium calcite grains and their subsequent precipitation as low-magnesium calcite cements via a process, defined by James and Jones^9^ as mineralogy-driven diagenesis. This process occurs at a local scale with little carbonate either entering or leaving the system—meaning that the void ratio does not change significantly during the cementation process.

As cementation progresses, the specific gravity of the sediment decreases (Fig. 8D) reflecting the transformation of aragonite grains (G_s_ = 2.94, after Cole and Little^71^) into calcite cement (G_s_ = 2.7, after Cole and Little^71^). It is possible to observe slightly divergent trends between inner-shelf (Balla Balla, Eighty Mile Beach) and mid-shelf (Paw) intervals, possibly reflecting differences in grain mineralogy, as inner shelf intervals tend to contain less pristine aragonite grains^26,38^. In this regard, further geochemical analysis including element mapping may help better understand these changes. In any case, these results suggest that, for a given site, specific gravity can be used as a proxy for the level of mineralogy-driven meteoric cementation. This is similar to trends observed using data from Nolting et al.^72^ and Rashed et al.^68^, whereby the youngest and least cemented NSG-rich grainstone exhibits the highest specific gravity.

#### Microcrystalline cement

**Figure 9:**
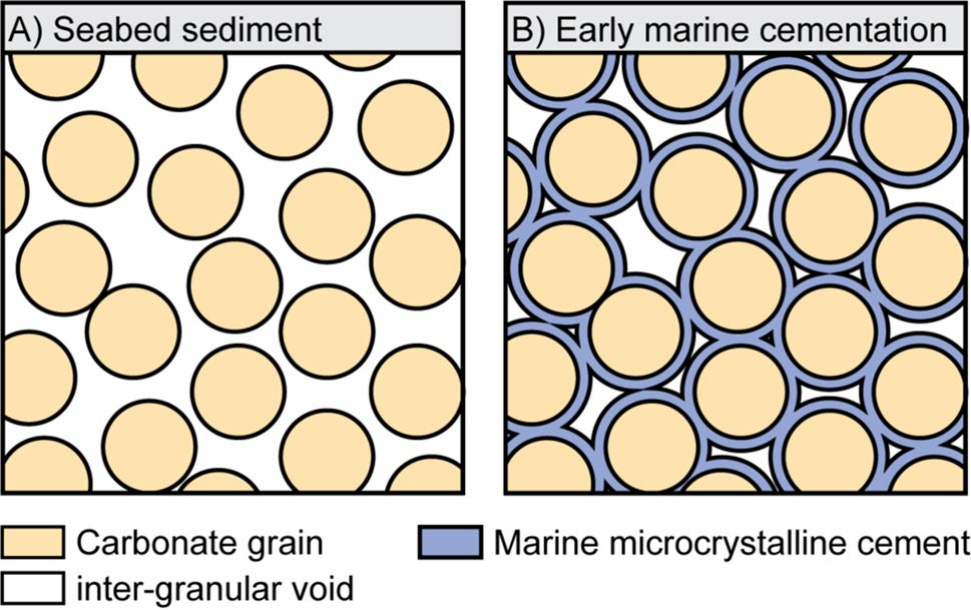
Summary sketch of the marine diagenesis observed in this study—With increasing diagenesis, seabed sediment (**A**) becomes surrounded with isopachous microcrystalline cement (**B**).

Microcrystalline cement was identified at Freycinet, consisting largely of microcrystalline rims (Fig. 9) and (to a lesser extent) of microcrystalline meniscus-like bridges, illustrating microbial activity in a marine subtidal environment^73^.

**Figure 10:**
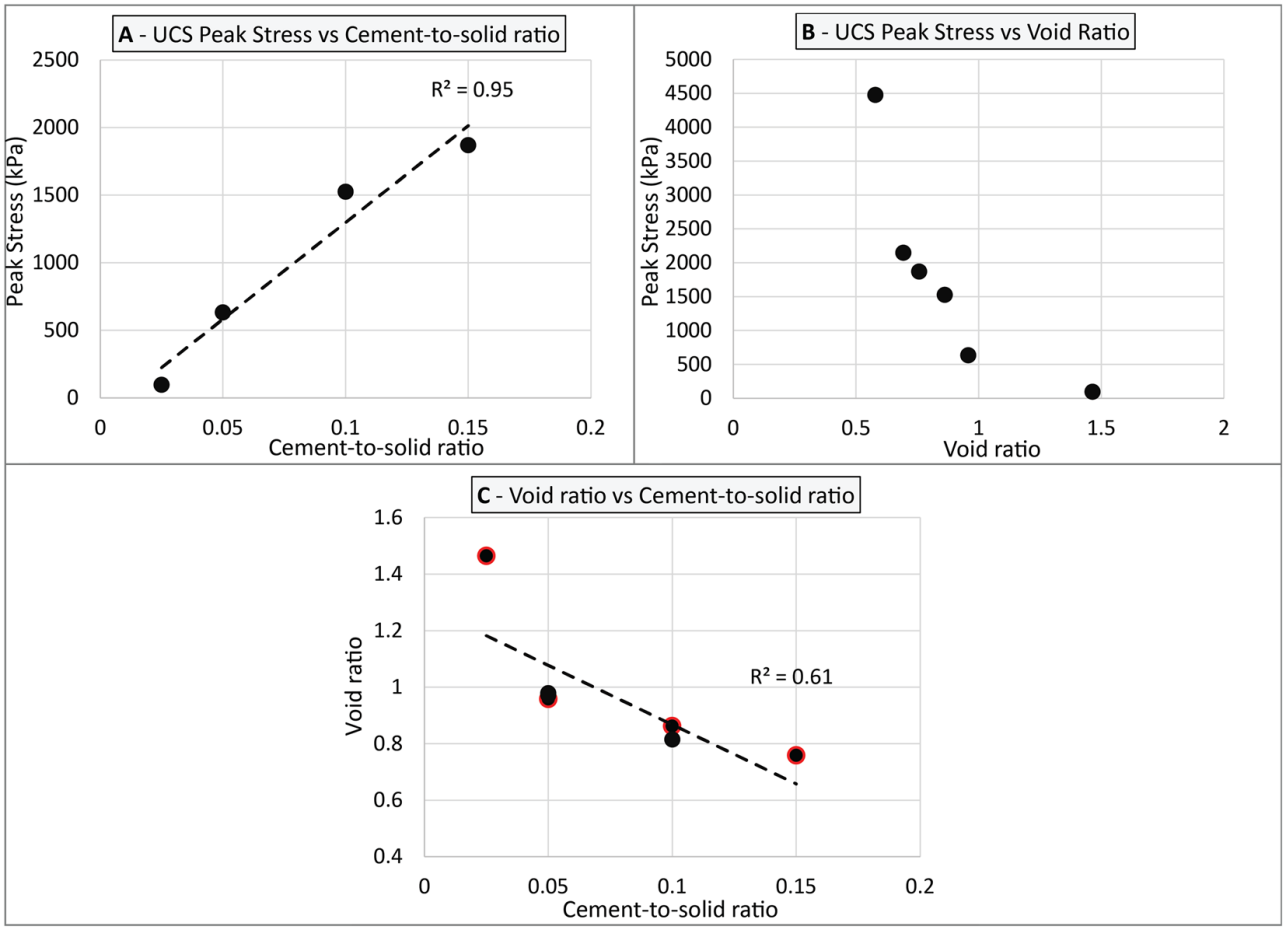
Properties of NSG-rich grainstone cemented as a result of marine diagenesis—(**A**) UCS Peak stress versus cement-to-solid ratio; (**B**) UCS Peak stress versus void ratio; (**C**) void ratio versus cement-to-solid ratio; Red labels indicate void ratio values that were calculated using dry density values from UCS tests.

UCS values increase with increasing cement-to-solid ratio and decreasing void ratio, suggesting there is a correlation between these parameters (Fig. 10A,B). Such behaviour is expected in tropical environments, where marine cementation results from the circulation of carbonate-saturated water in association with microbial activities^9,12,74^, meaning that the calcium carbonate fuelling early cementation originates from outside of the sediment. As a result, a decrease in the void ratio is associated with a proportional increase in the level of cementation (Fig. 10C), which in turn translates to an increase in the compressive strength (Fig. 10B). A relatively small number of grains are partially dissolved and associated with sparite infill, which may indicate a meteoric influence—indeed, samples were accumulated in a subtidal environment and may have experienced periods of exposure during subsequent sea-level fluctuation. Nevertheless, such meteoric influence appears somewhat limited, and is not expected to significantly alter observations drawn here on marine microcrystalline cements.

#### Differences between sparite and microcrystalline cement

**Figure 11:**
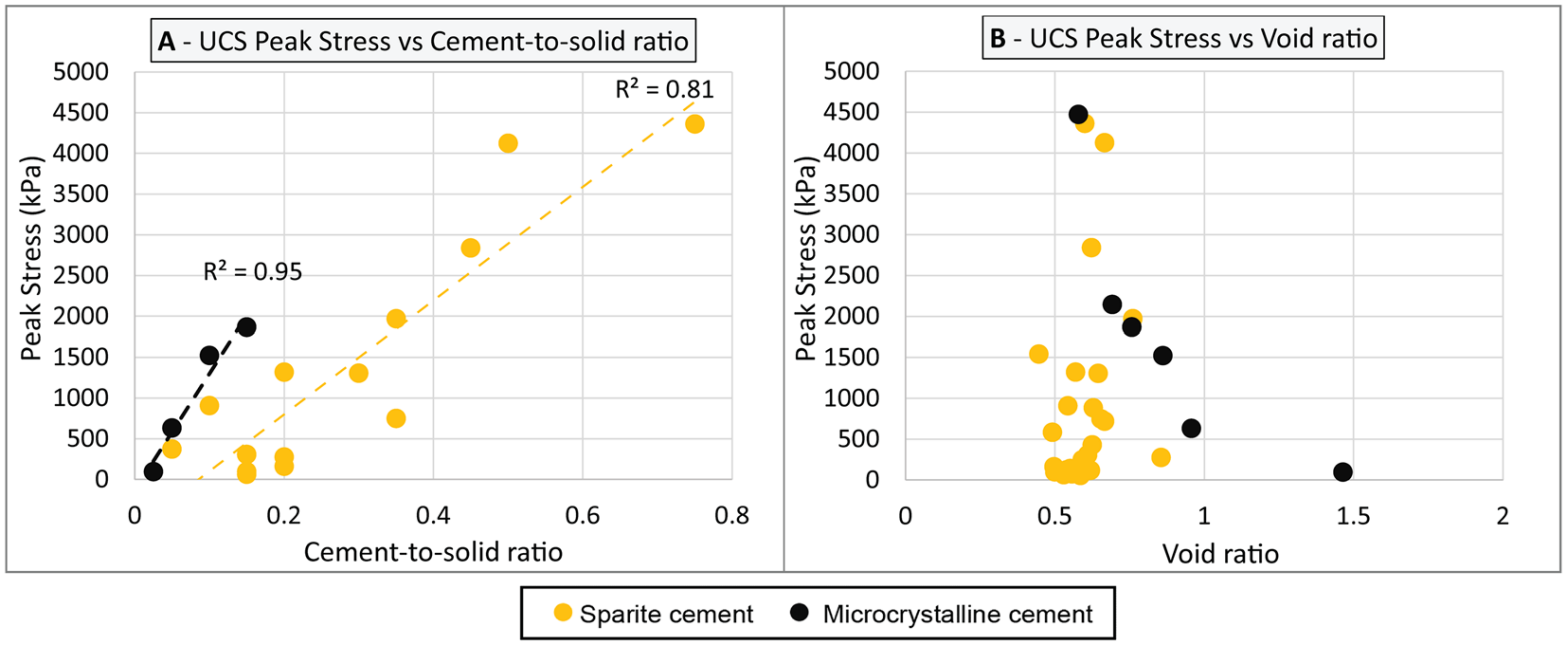
Comparison of NSGs-rich grainstone properties depending on the type of cement—(**A**) UCS Peak stress versus cement-to-solid ratio; (**B**) UCS Peak stress versus void ratio; Details on the source of the sparite cement samples presented in Fig. 8.

Overall, the data suggest that microcrystalline cements result in higher compressive strength values than sparite cements. Indeed, the Freycinet interval (comprising mainly microcrystalline cements) shows higher compressive strength than any of the other intervals, while exhibiting similar cement-to-grain ratio and void ratio values (Fig. 11A,B). While this interpretation is based on visual (and hence subjective) description of a limited number of samples, it is supported by previous observations from Nolting et al.^72^ and Rashed et al.^68^. This interpretation is further supported by lower strain and higher Young’s modulus values recorded in intervals cemented with microcrystalline calcite, suggesting that this is not a sampling bias (Table 3).

The physical process that causes the difference in compressive strength observed between microcrystalline and sparite cements is not fully understood. However, it is possible that heterogeneities associated with mineralogy-driven meteoric diagenesis, such as local variations in the level of cementation, result in development of weakened layers that facilitate premature failure of the material (when tested for UCS). Relative softness of sparite-bearing intervals would therefore reflect the distribution of the cement, rather than the strength of the cement itself. That said, it is likely that the morphology and size of the crystals also affects the compressive strength—considering that such a difference in compressive strength has also been observed in studies on burial diagenesis, where cement fills pore space^75–78^, and is in line with previous observations from Lézin et al.^79^ and Saad et al.^80^.

### Influence of the duration of exposure of the sediment

The intervals presented in this study show a variable compressive strength that is associated with varying degrees of cementation. This is particularly striking when considering that the Paw 1, Paw 2, Balla Balla, Eighty Mile Beach and De Grey intervals all contain similar grains that were subject to meteoric diagenesis. This raises the question—what controls the level of meteoric cementation, and how does it impact the geotechnical properties of a carbonate interval?

**Figure 12:**
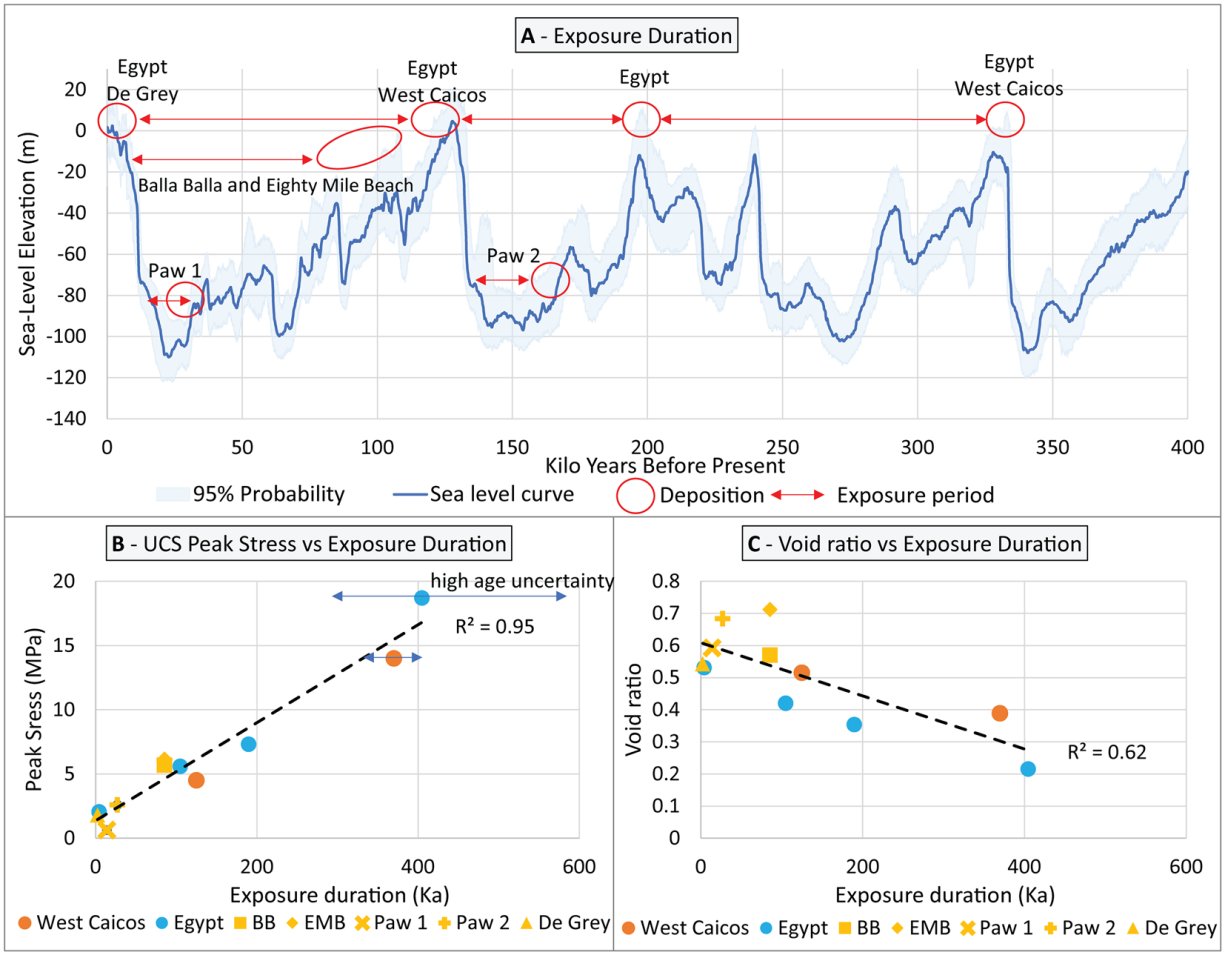
Comparison between the duration of meteoric cementation, the compressive strength and the void ratio using age data from Lebrec et al.^38^ and Lebrec et al.^26^—(**A**) illustration of the correction required to obtain the duration of exposure from Grant et al.^81^ RSL curve; (**B**) Average interval dry UCS Peak stress versus Exposure Duration, supplemented with data from Egypt^68,82,83^ and West Caicos^72^; (**C**) average interval void ratio versus age of deposition.

Using published age data from Lebrec et al.^26^, it is possible to investigate the compressive strength, and in turn the level of cementation, as a function of the age of each interval. However, given that meteoric diagenesis is only active above sea level, it is critical to correct the age of deposition to only retain the period during which the interval was above sea level (Fig. 12A). After applying a correction based on the relative-sea level curve from Grant et al.^81^, which had been previously deemed relevant for the NWS^25^, a pattern emerges whereby the longer an interval is exposed to meteoric diagenesis, the higher the compressive strength (Fig. 12B). The integration of case studies from West Caicos^72^ and Egypt^68,82,83^ further corroborates this hypothesis, while a similar (but not quantified) trend was also observed along the south-eastern Australian coast^84^.

### Influence of the local climate

Exposure time alone cannot explain all the differences observed in compressive strength. For instance, the De Grey sample has a compressive strength of about 1800 kPa but is associated with an exposure time of 2300 years, whereas the Paw 1 interval, which spent an estimated 14,000 years exposed to meteoric cementation, has an average compressive strength of only 300 kPa. Similarly, the level of cementation observed within the Paw 1 and Paw 2 intervals gradually decreases with depth and therefore with increasing stratigraphic age (Fig. 2D,F). Lastly, Despite being formed at similar times, some samples from Balla Balla and Eighty Mile Beach (BB5, EMB3) exhibit higher compressive strength—and lower void ratio—than others (BB4, EMB4).

Climate, and more specifically the amount of water flowing through the sediment and the depth of the water table, may provide an explanation for the above apparent inconsistencies. Indeed, Paw 1 was formed and subsequently exposed to meteoric diagenesis during the last glacial maximum, which was characterised by a much drier climate than the Holocene^45,85,86^, and so was exposed to a drier climate than the De Grey sample.

The gradual decrease in strength observed with depth at the Paw site could represent the infiltration of meteoric water from the top of the intervals which would have facilitated diagenesis. Likewise, the BB5 and EMB3 cores, which are associated with the highest strength values reported in this study, exhibit evidence of local water circulation (karsts, dissolution pipes) that are absent from the weaker BB4 and EMB4 cores.

These observations are in line with published literature on meteoric diagenesis^11,87^ with water flow facilitating carbonate minerals remobilisation. Such process, referred to as water-driven meteoric diagenesis (as opposed to the mineralogy-driven meteoric diagenesis, sensu James and Jones^9^) translates to increased cementation and compressive strength. The increased mobility of carbonate minerals throughout the system would then explain the reduction in void ratio observed at BB5 and EMB3 sites, as well as with increased exposure time in Egypt and West Caicos (Fig. 12C). Such a pattern is not observed in samples that underwent mineralogy-driven meteoric diagenesis only, highlighting the importance of this process.

Water-driven meteoric diagenesis may subsequently lead to the formation of calcrete layers and karsts^9,88^ resulting in significant heterogeneity within an interval. This is particularly visible at Balla Balla (and to a lesser extent at Eighty Mile Beach) where cores include thin calcrete crusts and karsts, including dissolution vugs, which are often associated with dissolution pipes and breccia (Fig. 4G). Dissolution features, in particular, may increase macroscale heterogeneity to an extent that cannot be captured by laboratory tests, given that the latter are performed on intact samples and therefore do not capture the effect of large vugs. The BB5 and EMB3 cores, for example, are located adjacent to open dissolution vugs that cut through the cores (i.e., the cores are in two halves, Figs. 4,G and 8D). In such a context, high compressive strength values measured on intact samples may not reflect the overall geotechnical behaviour of the interval.

At the other end of the spectrum, calcrete layers that develop along exposure surfaces and are commonly formed by the precipitation of microcrystalline cements^88^, would translate to significantly higher compressive strength values, further increasing the apparent heterogeneity. Such behaviour, while visually observed, could not be precisely measured here due to the limited thickness of the calcrete, but can be seen in data reported in West Caicos^72^ and Egypt^68^.

The Paw intervals which experienced much drier climatic conditions, and therefore underwent limited water-driven meteoric diagenesis, only show limited macroscale heterogeneity and little differences may be expected between laboratory and in-situ behaviour.

### Relevance to site characterisation

Being able to correlate the geotechnical properties of a sediment with its diagenetic environment, as well as their depositional environment^18,19^, can improve their predictability. Along the NWS, previous studies have defined identification criteria that discriminates between modern and relict features, as well as between now-submerged aeolian features and marine features^25^. On that basis, it is possible to determine from geophysical data alone whether an interval is likely to have undergone meteoric or marine diagenesis. For relict aeolianites, the level of cementation can be expressed as a function of the time of exposure and prevailing climate, which, considering that aeolianites were deposited during lower sea-level, can be related to water depth. Indeed, depending on their location (and water depth) on the shelf, now-submerged aeolianites would have spent more or less time above sea level and therefore exposed to meteoric diagenesis. It should be noted that, while not encountered here, special consideration should be given to post-depositional tectonic movement that may affect the duration of exposure.

Along the outer shelf, relict coastal dunes were formed under arid conditions during glacial periods^25^ and, due to their position on the shelf with respect to the relative sea level, have spent most of their time under water—therefore not exposed to meteoric diagenesis (Figs. 1B and 12A). As a result, these samples are expected to show moderate mineralogy-driven meteoric diagenesis and little-to-no water-driven meteoric diagenesis—resulting in a homogeneous, weakly cemented interval. Heterogeneity, when present, is likely to be expressed at a microscale, including, for example, variably cemented laminae (Fig. 7B).

On the other hand, samples from relict coastal dunes on the inner shelf were formed under wetter conditions^25^ and have spent much of their time exposed to meteoric diagenesis (Figs. 1B and 12A). This combination resulted in advanced mineralogy-driven diagenesis associated with varying degrees of water-driven diagenesis, leading to the development of significant macroscale heterogeneity related to the formation of dissolution vugs and calcrete layers (Fig. 7C).

Features identified at intermediate depths are expected to demonstrate a gradual transition from one extreme to the other. While this interpretation appears generally true based on available data, special care should be given to the stratigraphic relationship between sedimentary units, given that each successive eustatic cycle results in the development of additional coastal features, each associated with varying exposure time and in turn varying compressive strength. This is observed at the Paw Ridge, where Paw 2 exhibits a higher level of cementation and in turn of compressive strength than the younger Paw 1 ridge. Similarly, the modern shoreline coastal dunes are in the process of being formed and gradually bury the older coastal features. As a result, modern (loose) sediment coexists with slightly older and partly-cemented Holocene intervals, as well as with much older Pleistocene dunes that are expected to show similar properties to the Egyptian and West Caicos aeolianites.

The above discussion can only be readily made for aeolianites for which there is a relatively small body of data available across the shelf, albeit supported by other case studies across the globe^68,72,82,84^. The limited amount of data and published case studies that include both geological and geotechnical information is the main limitation and challenge for expanding this discussion and developing predictive models. Nevertheless, the results presented here, along with previously published studies^18,19^, show that the diagenetic and depositional environments can be related to geotechnical properties. This suggests that given sufficient opportunities to integrate all possible combinations of grain and cement types, it may be possible to further quantify the behaviour of the trends observed here (e.g., uncertainty, linear vs non-linear behaviour, boundaries, and ranges) and develop proper predictive models to support site characterisation. Such an approach requires establishing a comprehensive database, and it is therefore critical to encourage geotechnical researchers to investigate and present the geology of their samples. Doing so will help them to better understand the variability of their samples and also provide the basis for the development of quantitative predictive tools.

## Conclusion

Carbonate sediments are exposed to complex biological, chemical and mechanical processes, resulting in varying geotechnical behaviours that are challenging to predict. Recent studies have started to investigate the relationship between the genesis of carbonate grains and their geotechnical properties, with a particular focus on the internal architecture of the grains. Meanwhile, diagenetic controls on such properties have, to date, remained largely understudied. In that context, this study has integrated the petrographic descriptions, compressive strength and classification properties of six sedimentary intervals from the NWS of Australia that are composed of similar grains but with different diagenetic histories, to investigate how diagenesis affects the geotechnical properties of carbonate sediments.

The intervals are affected by either shallow marine or meteoric diagenesis. Shallow-marine diagenesis was observed within one interval (Freycinet) and is characterised by the formation of calcitic microcrystalline cement and infills. An increasing cement-to-solid ratio results in a linear reduction of the void ratio, in turn associated with an increase in the compressive strength of the material. On the contrary, mineralogy-driven meteoric diagenesis, which was observed at five locations (Paw1, Paw2, Balla Balla, Eighty Mile Beach and De Grey), is predominantly characterised by the  in-situ dissolution of aragonite grains and the subsequent precipitation of calcitic sparite cement. As a result, the void ratio is generally maintained throughout the process but evolves from mainly intergranular to intragranular (moldic). This transformation is accompanied by an increase in compressive strength and a decrease in the specific gravity. In any case, for a given cement-to-solid ratio, intervals cemented by microcrystalline cement exhibit higher compressive strength than those cemented by sparite cements.

The dataset enabled further investigation of the behaviour of carbonate sediment affected by meteoric diagenesis. First, it is shown that the compressive strength of carbonate sediments increases as a function of the time spent exposed to meteoric diagenesis, and, second, that the local climate affects the extent of the diagenesis, as well as the geotechnical behaviour of the sediment. Under arid conditions, meteoric diagenesis is minimal and largely controled by the mineral composition of the sediment, resulting in homogeneous, weakly cemented intervals. On the contrary, a wetter climate leads to increased diagenesis, associated with the formation of calcrete crusts and karsts. This results in highly heterogenous intervals that include both well-lithified sediment and weakened dissolved strata.

These results indicate that it should be possible to estimate the geotechnical properties of intervals affected by meteoric diagenesis with only limited site-specific data. Indeed, these intervals can be identified from geophysical data, while the regional climate can be known from the literature. Along the NWS, for example, such intervals were identified throughout the shelf and can be associated with either arid palaeo-environments along the outer shelf or wetter palaeo-environments along the inner shelf. As a result, the water depth, corrected for exposure time, can be used as a proxy to estimate their geotechnical behaviour. These results further suggest that, given enough work to investigate the relationship between the type of carbonate grains, diagenetic history and geotechnical properties, it should be possible to better forecast the properties of carbonate material that were so far considered problematic.

## Data Availability

All raw laboratory measurements are reported in the manuscript. Cores, samples and thin sections can be accessed on request at The University of Western Australia.
